# Idiopathic Acquired Dacryocystocele Presenting Only with Epiphora: A Very Rare Case Report

**DOI:** 10.7759/cureus.1653

**Published:** 2017-09-04

**Authors:** Muhammad Jahanzaib Anwar, Saad A Choudhry, Muhammad Aadil, Ahmer Asif, Atif Ameer

**Affiliations:** 1 Department of Internal Medicine, Rush University Medical Center; 2 Internal Medicine, University of California Davis; 3 Department of Medicine, FMH College of Medicine and Dentistry; 4 Department of Internal Medicine, FMH College of Medicine and Dentistry; 5 Department of internal Medicine, Lahore Medical And Dental College, Lahore, Pakistan

**Keywords:** idiopathic acquired dacryocystocele, epiphora, medial canthal swelling, dacryocystorhinostomy

## Abstract

A 42-years-old male presented with left medial canthal swelling, accompanied with only mild epiphora. There was no history of dacryocystitis, mid-facial trauma, surgery, or bloody tears. On examination, there was an immobile, subcutaneous, non-inflammatory mass below the medial canthal tendon. Lacrimal irrigation showed blockade at the nasolacrimal duct. A computerized axial tomography (CAT) scan revealed a non-enhancing, low-density, cystic lesion at the inferomedial margin of the orbit. There were no signs of bony erosion, consistent with idiopathic acquired dacryocystocele. The patient underwent external dacryocystorhinostomy (DCR) with silicone tube intubation. The patient’s symptoms of epiphora resolved after surgery. Idiopathic acquired dacryocystocele with only epiphora, although rare, should be considered in differential diagnosis of medial canthal, non-inflammatory swellings. In areas with insufficient endoscopic facilities, external dacryocystorhinostomy gives similar promising results.

## Introduction

An acquired dacryocystocele is a diffused enlargement of the lacrimal sac that forms as a result of a proximal or distal obstruction of the lacrimal drainage system. Swelling of the medial canthal region along with watery eyes are the most common symptoms. It is common in children but its incidence is very rare in the adults. Dacryocystocele is associated with irreversible fibrosis leading to the narrowing of the lacrimal system, ossification of the nasolacrimal duct, and low-grade inflammation [1]. Adult dacryocystocele typically happens as sequelae to dacryocystitis, sinusitis, paranasal sinus mucocele, after trauma or tumor, or secondarily to dacryocystorhinostomy surgeries; however, rarely, it may occur idiopathically or due to autoimmune fibrous diseases.

Radiographically, a dacryocystocele appears as a collection of fluid surrounded by a rim that separates it from the adjacent bony structures [2]. Diverticulum, dermoid and epidermoid cysts, encephalocele, malformations of the lacrimal sac, primary tumors of the sac, and external tumors must be ruled out before making the diagnosis of Idiopathic acquired dacryocystocele [1]. Idiopathic acquired dacryocystocele with only epiphora and without any associated dacryocystitis is a very rare disease. Only five cases have been previously discussed [3-8]. We are presenting another case of idiopathic acquired dacryocystocele associated only with epiphora.

## Case presentation

**Figure 1:**
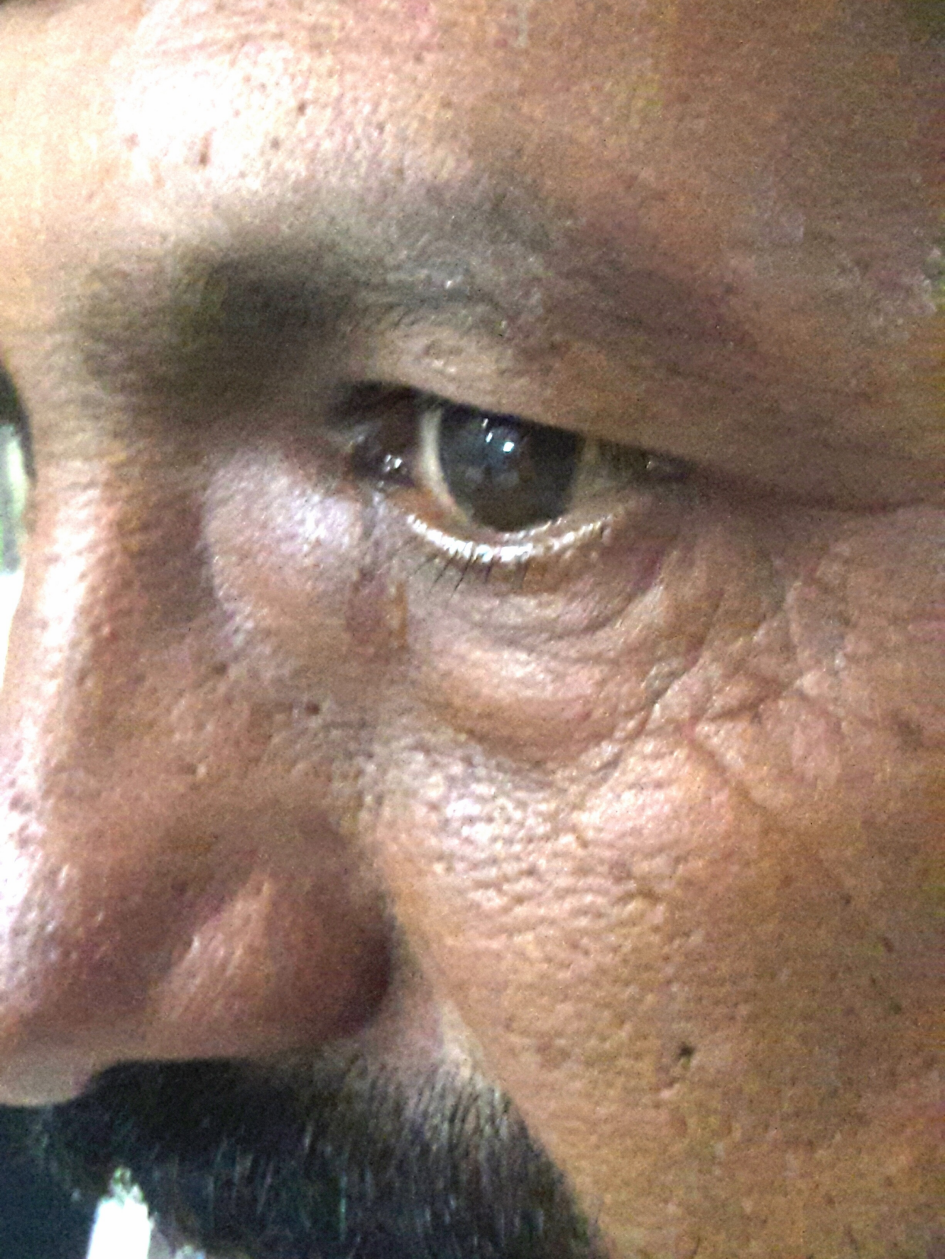
In a 42-year-old-man who had epiphora of the left eye, an immobile, subcutaneous and non-tender mass 2.0 cm in diameter was locatednear the left medial canthus.

A 42-year-old man presented with swelling at the medial canthus of the left eye associated with epiphora. The swelling appeared four years back and gradually increased in size. There was no history of dacryocystitis, mid-facial trauma, surgery, or bloody tears. On examination, there was an immobile, subcutaneous, noninflammatory mass below the left medial canthal tendon (Figure 1). Regurgitation test was negative on the left side. There was a delay in dye disappearance and lacrimal irrigation showed blockade at the nasolacrimal duct. An otorhinolaryngology consult and examination did not show any nasal cyst or polyp and there was no evidence of paranasal sinusitis.

**Figure 2:**
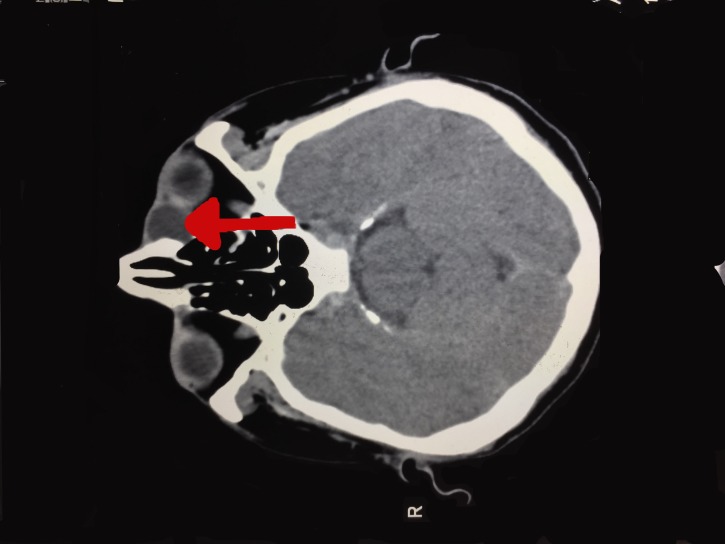
CAT scan of the skull and orbit showing non-enhancing, low-density, cystic lesion at the inferomedial margin of the left orbit (red arrow) CAT: computerized axial tomography

A computerized axial tomography (CAT) scan revealed a 2.0 x 2.0 cm non-enhancing, low-density, cystic lesion at the inferomedial margin of the left orbit. There were no signs of bony erosion consistent with idiopathic acquired dacryocystocele (Figure 2).

The patient was treated conservatively with topical antibiotics and sac massage for one week, but there was no improvement. The patient underwent external dacryocystorhinostomy (DCR) with silicone tube intubation. During the surgery, a clear, yellowish fluid was drained from the lacrimal sac. The lacrimal sac was biopsied and the fluid was sent for culture. The culture of the fluid was negative and histopathology of the sac showed a squamous epithelium with goblet cells. There were no neoplastic cells, confirming the diagnosis of idiopathic acquired dacryocystocele.

The patient’s symptoms of epiphora resolved right after the surgery. The silicone tube is in place and it will be removed after six months.

## Discussion

Acquired dacryocystocele, contrary to its congenital counterpart, occurs solely in adults and is a very rare condition [4]. To label a case as idiopathic acquired dacryocystocele, secondary causes of acquired dacryocystocele, i.e., chronic infections, trauma, primary and secondary tumors, and idiopathic blockade of nasolacrimal duct must be ruled out [4-5]. The present case of acquired dacryocystocele did not have any secondary causes, so it is most likely idiopathic. Chronic nasolacrimal duct obstruction and the resulting secondary functional proximal obstruction at the junction of the canaliculus and the sac results in the formation of idiopathic acquired dacryocystocele.

Dacryocystitis is a common complication that can occur with idiopathic acquired dacryocystitis. It was present in all patients in two case series conducted by Woo K, et al. and Xiao MY, et al. [5-6]. Epiphora as the only symptom without accompanying dacryocystitis is very rare. Only five cases of idiopathic acquired dacryocystocele with only associated epiphora have been previously reported [3-8].

Conservative treatment with eye drops, lacrimal sac massage, and probing of nasolacrimal duct relieves the occlusion in congenital dacryocystocele, but in adults, surgical correction is the mainstay of treatment due to different pathogenesis [4]. Dacryocystorhinostomy (DCR) is the treatment of choice for acquired dacryocystocele. Endonasal endoscopic DCR is preferred over external DCR due to the possibility of avoiding a facial scar, less bleeding, faster rehabilitation, and to prevent the pump function of the orbicularis oculi muscle [9]. But external and endoscopic approaches have similar success rates, so in areas with insufficient endoscopic facilities, external DCR gives similar promising results [9].

## Conclusions

Although very rare, idiopathic acquired dacryocystocele should be kept as a differential diagnosis of non-inflammatory medial canthal swelling. Uncomplicated idiopathic acquired dacryocystocele presenting only with epipohora is an extremely rare condition that responds to dacryocystorhinostomy very effectively.
